# Varied overground walking-task practice versus body-weight-supported treadmill training in ambulatory adults within one year of stroke: a randomized controlled trial protocol

**DOI:** 10.1186/1471-2377-11-129

**Published:** 2011-10-21

**Authors:** Vincent G DePaul, Laurie R Wishart, Julie Richardson, Timothy D Lee, Lehana Thabane

**Affiliations:** 1 School of Rehabilitation Sciences, McMaster University, Hamilton, Ontario, Canada; 2 Department of Kinesiology, McMaster University, Hamilton, Ontario, Canada; 3 Department of Clinical Epidemiology and Biostatistics, McMaster University, Hamilton, Ontario, Canada; 4 Biostatistics Unit, Father Sean O'Sullivan Research Centre, St Joseph's Healthcare - Hamilton, Hamilton Ontario, Canada; 5 Physiotherapy Department, St. Joseph's Healthcare Hamilton, Hamilton, Ontario, Canada

## Abstract

**Background:**

Although task-oriented training has been shown to improve walking outcomes after stroke, it is not yet clear whether one task-oriented approach is superior to another. The purpose of this study is to compare the effectiveness of the Motor Learning Walking Program (MLWP), a varied overground walking task program consistent with key motor learning principles, to body-weight-supported treadmill training (BWSTT) in community-dwelling, ambulatory, adults within 1 year of stroke.

**Methods/Design:**

A parallel, randomized controlled trial with stratification by baseline gait speed will be conducted. Allocation will be controlled by a central randomization service and participants will be allocated to the two active intervention groups (1:1) using a permuted block randomization process. Seventy participants will be assigned to one of two 15-session training programs. In MLWP, one physiotherapist will supervise practice of various overground walking tasks. Instructions, feedback, and guidance will be provided in a manner that facilitates self-evaluation and problem solving. In BWSTT, training will emphasize repetition of the normal gait cycle while supported over a treadmill, assisted by up to three physiotherapists. Outcomes will be assessed by a blinded assessor at baseline, post-intervention and at 2-month follow-up. The primary outcome will be post-intervention comfortable gait speed. Secondary outcomes include fast gait speed, walking endurance, balance self-efficacy, participation in community mobility, health-related quality of life, and goal attainment. Groups will be compared using analysis of covariance with baseline gait speed strata as the single covariate. Intention-to-treat analysis will be used.

**Discussion:**

In order to direct clinicians, patients, and other health decision-makers, there is a need for a head-to-head comparison of different approaches to active, task-related walking training after stroke. We hypothesize that outcomes will be optimized through the application of a task-related training program that is consistent with key motor learning principles related to practice, guidance and feedback.

**Trial Registration:**

ClinicalTrials.gov # NCT00561405

## Background

Every year an estimated 9 million new stroke events occur globally, and an additional 30.7 million individuals live with the ongoing effects of stroke [1]. Decreased ability to walk is one of the most common and debilitating functional limitations following stroke [2-4]. Although the majority of adults with history of stroke recover some ability to ambulate independently following rehabilitation [2], many individuals experience long term residual limitations in walking speed [5,6], endurance [6] and walking-related self-efficacy [7,8]. Between 27% and 50% of community dwelling individuals report difficulty walking outside of their homes for months and years following stroke onset [6,9-11]. In the face of these difficulties, independent walking remains one of the most frequently-stated goals of stroke rehabilitation [12], with 75% of individuals identifying the ability to walk in the community as a priority in living at home [10]. Given these challenges, stroke-rehabilitation clinicians and researchers are compelled to apply and evaluate interventions that optimize the recovery of walking skill and participation in community mobility related activities.

According to recent stroke-rehabilitation reviews and practice guidelines, optimal walking recovery may be realized through the application of a task-related walking training approach [13-15]. In the literature, the term task-related walking practice generally refers to any intervention where walking or walking-related tasks are practiced using a functional approach [16]. Alternate terms include task-specific [13,17], task-oriented [18,19], and task practice [14,20,21]. Although the specific content of interventions varies, they are all based on the premise that in order to optimally improve walking skill, one must practice walking. Training protocols include walking tasks performed overground, on a treadmill, or both. Two of the most common interventions described in the stroke-rehabilitation literature include practice of a variety of primarily overground walking-related tasks [16], and body-weight-supported treadmill training (BWSTT) [22].

### Varied Overground Walking-task Training

Rooted in movement science, including motor learning research, Carr and Shepherd were early advocates of task-related walking practice after stroke [23]. They emphasized the importance of patient engagement in abundant, active practice of the whole task of walking. In addition, they promoted the practice of varied walking-related tasks organized in a circuit of stations. A small number of controlled studies have evaluated the effectiveness of this varied task practice approach in community-dwelling adults with stroke history [19,24,25]. These studies differ in quality, intervention content and effect on walking performance. In a small-sample pilot study by Dean and colleagues [24], 12 individuals with chronic stroke were randomized to a varied task-related training protocol, including overground walking, treadmill walking, and walking-related tasks (e.g. heel raises, step-ups, narrow base standing), or to a control intervention (upper- extremity task training). The experimental group improved walking endurance and speed more than the control group, however, the authors failed to discuss the implications of the relatively high proportion of participants who did not complete the study (n = 3). In a larger trial, 91 individuals within one year of stroke were randomized to receive 18 sessions of varied walking-related task practice, or upper-extremity task practice performed in sitting [19]. The experimental intervention included practice of walking tasks (i.e. stand up and walk, walking along a balance beam, walking backwards, walking while carrying, walking with speed, stairs and walking on a treadmill) and walking-related tasks (i.e. step-ups, kicking a ball). Following treatment, the walking group demonstrated significantly greater changes on the 6-minute walk test [35 m more than control, 95% confidence interval (CI) 7, 64], gait speed (0.11 m/s more than control, 95% CI 0.03, 0.19) and walking-related self-efficacy. In a more recent trial, 58 adults with chronic stroke were assigned to a 12-session walking-related task training protocol or to a non-exercise control intervention [25]. In this study, only 4 of the 15 stations involved walking while the remaining stations focused on strength and balance tasks in standing or sitting. The authors reported that the experimental group demonstrated modest, but statistically greater gains on the 6-minute walk test (19 m, p = 0.03) compared to the control group.

Based on this literature, variable practice of walking and walking-related activities in a circuit format is associated with greater improvements in gait speed, endurance and walking self-efficacy than a non-walking control intervention such as upper-extremity task practice. To date we do not know if this approach is superior to an alternate walking-focused treatment.

### Body Weight Supported Treadmill Training

BWSTT is rooted in the central pattern generator (CPG) theory of gait control and recovery [26]. The theory proposes that gait is largely controlled by a set of neurons located primarily at the spinal level [27], and these CPG's can be activated through the afferent input associated with typical gait through passive or assisted limb movements, weight shift, and postural alignment [26-28]. Mass repetition of these movements is thought to result in neural reorganization and subsequently improve capacity for over-ground walking in individuals with history of stroke [29,30]. As described in the literature, BWSTT requires the use of specialized body weight support equipment, a treadmill and the assistance of one to three trainers [22]. While recommended in opinion papers and reviews [31,32], when planning our study we found only 3 controlled trials that have evaluated the effectiveness of BWSTT in community dwelling individuals post-stroke [17,33,34]. In 2002, Sullivan randomized 24 individuals with chronic stroke to one of three BWSTT protocols; fast speed, variable speed and slower speed [33]. After 12 sessions, participants who trained at fast speeds improved overground velocity by 0.08 m/s more than those who trained at slow speeds (p = 0.04). In a larger RCT, 80 individuals with chronic stroke were assigned to one of the four combined treatment protocols; BWSTT and arm ergometer, cycling and arm ergometer, BWSTT and lower extremity strength training, and BWSTT and cycling [34]. The group that received alternating sessions of BWSTT and arm-ergometer exercise (12 sessions each over 6 weeks) improved overground gait speed by 0.12 m/s (p < 0.01) more than those who received the cycling and arm-ergometer program. There were no significant differences between the change scores of the different BWSTT interventions. Finally, in a recent repeated-measures, randomized crossover study [17], 20 adults with chronic stroke and recently discharged from physical therapy were assigned to receive either 12 sessions of BWSTT followed by 4 weeks of no treatment, or 4 weeks of no treatment then 12 sessions of BWSTT. Improvements in gait speed, gait efficiency (O_2 _cost) and daily stepping activity were observed after BWSTT treatment periods and not following the no-treatment periods. Based on these small-sample controlled trials, approximately 12 sessions of BWSTT seems to be more effective than a no-treatment control intervention or a non-walking intervention such as cycling. In addition, improvements seem to be optimized when participants train at speeds greater than their typical overground walking speeds. To date the effectiveness of BWSTT has not been evaluated against an alternate program of overground walking-focused training in community dwelling adults with history of stroke.

In summary, varied overground-focused walking practice and BWSTT have been shown to result in greater improvements in walking speed, endurance and/or self-efficacy when compared to non-walking interventions (i.e. arm and hand exercises, cycling). These two walking-task related interventions are different in theoretical rationale as well as in content. In the case of BWSTT, the rationale is clear - repetition of the normal stepping pattern of gait activates the locomotor CPG's and results in improved overground walking. Practice is constant and blocked, guidance is provided liberally, and the use of the treadmill environment allows for the repetition of a more normal gait pattern thought to be necessary to activating the CPG's [17,33,34]. In varied overground-focused walking practice, the theoretical premises for learning are less defined. While all studies implicitly apply the motor learning principle of specificity of practice, these overground-focused walking task training interventions fail to take full advantage of decades of behavioral motor learning research that have identified optimal learning conditions in healthy adult and rehabilitation populations [35]. For example, based on this research, retention and transfer of learned skills are typically enhanced if practice is abundant, variable, and organized in a random rather than blocked order. Learning is typically optimized if augmented feedback is delayed and intermittent rather than immediate and continuous and if physical guidance is not excessive but allows learners to experience and attempt to correct their own errors. Although the overground-focused task-related training interventions include variable practice of walking tasks that resemble typical walking conditions, order of practice is blocked, and feedback schedule is not described [19,24,25]. We suggest that the impact of task-related walking training will be more fully realized if the content and structure of interventions are consistent with these key motor learning principles.

The purpose of this randomized controlled trial is to compare the impact of the Motor Learning Walking Program (MLWP), a 15-session program of varied overground walking-task training consistent with key motor learning principles related to practice, guidance and feedback, to 15 sessions of BWSTT on walking performance in community-dwelling, ambulatory adults within 12 months of stroke onset.

It is our hypothesis that participants assigned to the MLWP group will demonstrate greater scores in comfortable gait speed and secondary outcome measures at post intervention and follow-up assessments.

## Methods/design

### Design Outline

**Figure 1:**
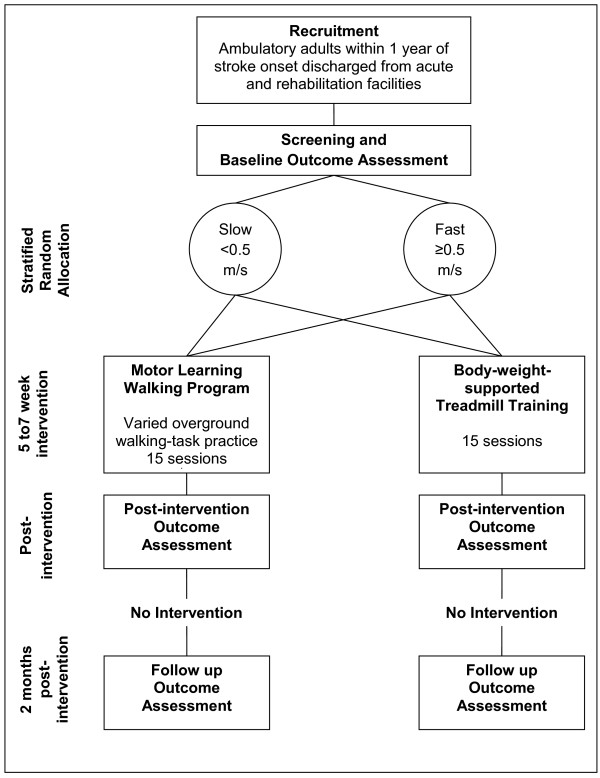
**Study design and timelines**.

This study is a prospective, randomized, single blind, balanced parallel-group (1:1) superiority trial with stratification by baseline comfortable gait speed (< 0.5 m/s and ≥ 0.5 m/s). The design includes concealed allocation during recruitment and screening, blinded outcome assessment and intention to treat analysis. Refer to Figure 1 for study design diagram.

### Ethics

All study activities have been approved by the Research Ethics Boards of St. Joseph's Healthcare Hamilton (#6-2753), the Hamilton Health Sciences/Faculty of Health Sciences McMaster University (#07-054), and Joseph Brant Memorial Hospital, Burlington, Ontario.

### Participants

The target population of this trial is community-dwelling, ambulatory older adults with mild to moderate stroke-related walking dysfunction within twelve months of most recent stroke onset. In contrast to most previous trials [17,24,25,33,34], time since onset was limited to less than one year as it represents the period when patients are most likely to access community-based rehabilitation interventions. Seventy participants will be recruited from clients about to be discharged from inpatient acute and rehabilitation units and outpatient programs at two teaching hospitals in Hamilton, Ontario, Canada (St. Joseph's Healthcare Hamilton and Hamilton Health Sciences) and one community hospital, Joseph Brant Memorial Hospital, in the neighbouring community of Burlington. We expect that treating physiotherapists and other clinicians will refer the majority of potential participants; however, some individuals may self-refer in response to community advertisements. Following screening, individuals will be invited to participate if they meet the following criteria: 1) living in the community at time of entry into study, 2) at least 40 years old, 3) within 12 months of onset of a physician diagnosed ischemic or hemorrhagic stroke in any brain location (with or without evidence from diagnostic imaging), 4) able to walk 10 m without assistance with self-selected gait speed < 1.0 m/s (or typically use a walking aid), 5) able to follow a 2-step verbal command, 6) independent with community ambulation prior to most recent stroke, and 7) receive physician approval to participate in the study. Individuals with history of more than one stroke who meet all other inclusion criteria will be included in the study. Individuals will be excluded if they present with: 1) marked cognitive impairment (i.e. Mini Mental Status Exam < 24/30 or score less than predicted according to age and education level) [36], 2) severe visual impairment, 3) lower extremity amputation, 4) presence of serious unstable cardiac, medical or musculoskeletal conditions that would limit safe participation in walking exercise (as determined by physician screening and baseline assessment interview).

### Randomization

Participants will be randomly allocated to the two active intervention groups using a fixed allocation ratio of 1:1. Consistent with previous studies in this area [19,33], we anticipate the response to both training programs to be associated with pre-treatment walking ability and participants will be stratified by baseline comfortable gait speed (slow < 0.5 m/s and fast ≥ 0.5 m/) to minimize group imbalances on this variable [37]. In order to maintain recruitment balance between groups throughout the trial, a permuted block randomization process will be used within each strata using block sizes of at least 2 with all blocks divisible by 2 [38]. The randomization creation process (including block sizes) and resulting schedule will be set, held and managed by a central randomization service (Biostatistics Unit at St. Joseph's Healthcare Hamilton). Group assignment will be communicated by email to the research coordinator on a single participant basis after screening, written informed consent, and baseline assessments have been completed.

### Experimental Intervention: Motor Learning Walking Program (MLWP)

The Motor Learning Walking Program is a program of varied overground walking-task practice based on theory and research from the fields of motor control, motor learning, neuroplasticity, and stroke rehabilitation. The following statements will be used to guide the implementation of the MLWP:

***1. Motor skill is the product of multiple systems, internal and external to the individual ***[39]. Skilled human walking arises from the distributed contribution of both internal (e.g., musculoskeletal, cardiovascular and central nervous system) and external systems (e.g. the environment). The characteristics of walking will vary depending on the specific task and environmental context in which it is performed. A comprehensive rehabilitation program must address the known demands of community walking [40].

***2. Learning is defined as a relatively permanent change in skill level (retention) and the ability to perform skill under varied conditions (transfer) ***[35]. ***Motor learning is typically specific to the conditions of practice***. Practice conditions should resemble the conditions of expected typical performance, including task characteristics, sensory motor conditions and information processing demands [35]. Repetitive task oriented practice of walking results in improved walking outcomes after stroke [20]. Training-induced neuroplasticity is specific to the trained movement or skill [41,42].

***3. Practice should be sufficiently intense***. Increased amounts of practice (repetitions) are typically associated with increased learning [35,42]. Increased practice of lower extremity focused activities is associated with improved recovery of walking after stroke [43].

***4. Practice must be sufficiently challenging and engaging***. Motor learning is enhanced when the learner is cognitively challenged during practice or training [44,45]. Cognitive effort may be facilitated through non-repetitive (random or serial) practice schedule, opportunity for self-evaluation and error correction through reduced augmented feedback presentation and minimal physical guidance, and increased task complexity [44-46]. Motor learning rather than simple motor activity or movement repetition is required to induce cortical and sub-cortical reorganization [42,46]. Practice must be interesting, meaningful, with the learner/client actively engaged in order to induce desired neuroplastic changes [42].

***5. Variable practice optimizes learning***. Practice of a skill under a variety of environmental and task conditions usually leads to improved retention and transfer of skill to novel performance conditions [35].

***6. The effect of variable practice is usually enhanced when practiced in a non-repetitive order ***[35].

### Content of the MLWP

**Figure 2:**
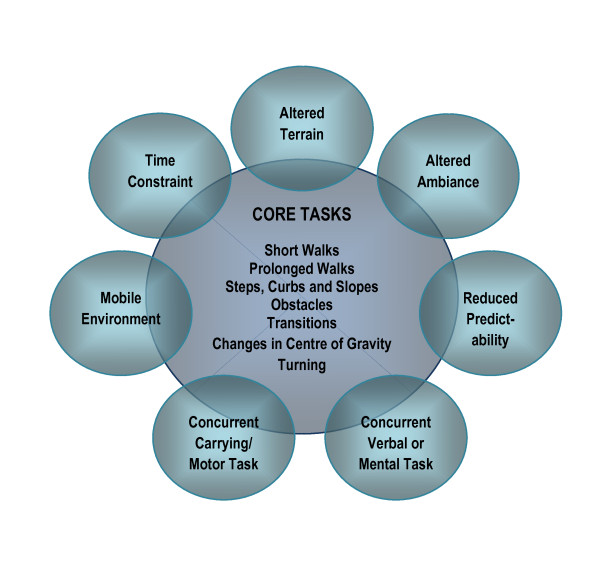
**Motor Learning Walking Program**. Every session includes all seven core tasks described in the centre circle. During or between sessions, the training physiotherapist may adjust the level of challenge of each core task by adding or removing one or more of the task complexity factors described in the outside circles.

At the first session, the therapist will spend 15 minutes to establish walking-related goals with the client. These goals will help inform the content and emphasis of the walking training program. Training will be organized to promote engagement in intense, repetitive practice of a variety of challenging, walking tasks. Practice will be cognitively effortful, encouraging participants to solve and re-solve the problems of walking in a variety of environmental and task conditions. Refer to Figure 2 for a graphic representation of the MLWP.

#### Core Tasks

Participants will practice all walking tasks overground. At every session, the therapist will incorporate the following seven core tasks that reflect the typical demands of home and community ambulation [40,47]: 1) walk short distances, 2) walk prolonged distances or times (> 50 m or > 5 minutes), 3) steps, curbs and slopes, 4) obstacle avoidance, 5) transitional movements (e.g. sit to stand and walk), 6) changes in centre of gravity (e.g. pick up object from floor while walking) and, 7) changing direction/turning while walking.

#### Increasing Complexity of Walking Task Practice

Using the concepts described by Gentile in her Taxonomy of Task Analysis [48] the training therapist will make each of the core tasks more complex through the addition of concurrent mental, verbal or physical tasks, adding a time restraint, altered terrain and/or lighting, increased duration, reduced predictability and/or performance of walking in a mobile environment.

The therapist will adjust the difficulty of practice tasks based on their assessment of the participant's ability to perform the task safely without maximum physical assistance.

Tasks will be practiced in a serial or random order, moving from task to task, avoiding repetition of one station more than two times in a row. Feedback will be delayed and participants will be asked to self-evaluate their performance on a task and develop strategies to improve performance. When feedback is given, it will include either knowledge of results (e.g. time taken to complete a specific task) or knowledge of performance (e.g. step length, stance time) types of feedback [49]. The therapist will only provide hands-on guidance or assistance when required for safety, or for initial completion of the basic task. Specific handling or facilitation techniques will not be used to affect quality of gait. Participants will practice walking tasks with and without their preferred gait aid. Tasks will be practiced in the physiotherapy gym and/or more natural settings inside and outside the hospital (e.g. courtyard, sidewalks, hospital lobby). The tasks will be designed to encourage inclusion of both lower limbs during practice (e.g. reciprocal stepping up stairs). Each session will last 45 minutes including intermittent rest periods as required. Participants will practice three times a week for five weeks for fifteen sessions. Refer to Figure 2 for a graphic representation of the Motor Learning Walking Program.

### Comparison Intervention: Body Weight Supported Treadmill Training (BWSTT)

Participants in the control group will practice walking on a treadmill according to a protocol based on an intervention described by Sullivan et al. [33] and Duncan et al. [50]. Based in the CPG theory of stepping control and recovery [51], the focus of this intervention is to provide participants with an opportunity to practice many repetitions of the normal gait cycle. Within a 45-minute session, participants will practice walking for up to 30 minutes at a time on the treadmill. Participants will train using the LiteGait system (harness and mechanical overhead suspension) and the GaitKeeper treadmill (Mobility Research Inc.). All participants will initiate training with 30% of their total body weight supported. A maximum of 40% body weight support will be provided during training. As recommended in the literature [33,52], participants will practice walking on the treadmill at speeds above their preferred overground walking speeds, preferably at or above 0.89 m/s (or 2.0 mph). Physical guidance will be provided by 1 to 3 therapists at the participant's pelvis, and/or their limbs to increase gait symmetry, facilitate weight shift, increase hip extension during stance, and to correct foot placement. Verbal feedback related to the participants gait pattern (knowledge of performance) will be provided frequently and concurrent to participants walking on the treadmill. Continuous visual feedback will also be provided via a full-length mirror. Participants will be discouraged from placing their hands on the LiteGait or treadmill handles during training. Body weight support, feedback, and guidance will be weaned, and treadmill speed adjusted according to a clinical decision making algorithm modified from a training algorithm described for individuals with spinal cord injury by Behrman et al. [53]. A comparison of key elements of the MLWP and BWSTT are provided in Table 1.

**Table 1:** Description of experimental and comparison interventions

Learning Variable/Principle	Motor Learning Walking Program	Body Weight Supported Treadmill Training
**Amount of Practice/Intensity**	Up to 40 minutes of walking activity per session 15 sessions over 5 weeks	Up to 30 minutes treadmill walking per session 15 sessions over 5 weeks
**Specificity Of Practice**	Reflects task and environmental demands of community walking	High repetitions of near normal gait cycle on treadmill
**Variable Practice**	Variable practice of different overground walking tasks	Single task practice - walking on treadmill
**Practice Order**	Random or serial order, moving through different tasks returning to each task at least once.	Blocked or mass practice of single task of walking on treadmill
**Augmented** **Feedback**	Encourage self-evaluation through delayed, intermittent and summary feedback KP and results KR provided	Continuous, immediate visual (mirror) and/or verbal feedback. Focus on KP, specifically related to posture and gait pattern
**Instructions**	Instructions provided related to the goals of the task. Emphasis on problem solving, discovery of alternate ways to complete walking tasks.	Instructions regarding performance of near normal gait pattern
**Physical Guidance**	Physical guidance provided for safety, or initial completion of basic task early in learning. Emphasis on allowing participants to make and attempt to correct errors.	Frequent guidance of one to three trainers at pelvis, hemi and non-hemi-limb to guide position and timing Up to 40% body weight support provided through harness - weaned according to performance Handle use discouraged Errors prevented or minimized
**Training Personnel**	Physiotherapist × 1	Physiotherapist × 1 plus 1 to 2 other physiotherapists or physiotherapy assistants
**Training Setting**	In hospital physiotherapy department, other parts of hospital and outdoors	In hospital outpatient department on treadmill
**Training Speed**	Practice of comfortable and fast walking	Will train at, or above target speed 2.0 mph (0.89 m/s) as soon as participant is able
**Use Of Walking Aid/Orthoses**	Practice with and without orthoses and walking aid	Practice without walking aid, may use orthoses if necessary

KP = knowledge of performance, KR = knowledge of results

For experimental and control interventions, blood pressure (BP), heart rate (HR) and rating of perceived exertion (RPE) will be measured at the beginning, during rest periods and at the end of every treatment session. During training, exercise intensity will be reduced if HR exceeds 70% of age predicted maximal heart rate (220 - age) or RPE is greater than 13 on the Borg RPE scale. If resting BP exceeds 180 mmHg systolic and/or 100 mmHg diastolic, the exercise session will be stopped and their physician notified. This information will be recorded allowing comparison between groups. Patients will also wear the StepWatch 3^© ^step activity monitor during training sessions, and mean number of steps taken during the sessions recorded as a measure of amount of task-related practice.

In the event of missed sessions, participants will be allowed a maximum of seven consecutive weeks to complete as many of the fifteen sessions as possible. Considering that previous studies have demonstrated changes in walking skill following 12 [24,25,33,34] and 18 sessions [19] over 4 [24,25,33,35] to 6 [19,34] weeks, we expect that a training frequency of 2 to 3 sessions per week for a total of 15 sessions will be result in improved walking skill in our participants.

To minimize the risk of contamination, separate training physiotherapists will deliver the Motor Learning Walking Program and the BWSTT program. All therapists will undergo a standardized training program prior to treating study participants on their own. The principal investigator will monitor ongoing competence and adherence through session observation, case discussions and documentation reviews. In order to minimize the impact of expectation bias, training therapists and participants will be blinded to the hypotheses of the investigators regarding which of the two interventions is expected to result in superior outcomes. To avoid co-intervention, participants will be asked to refrain from attending physiotherapy for their balance or walking limitations during the study intervention period. Participants will be questioned at post-intervention and follow up measures regarding their participation in physiotherapy outside of the study.

### Outcomes

Efficacy of the interventions will be determined by comparing change scores (baseline to post treatment) on a variety of standardized outcome measures taken at baseline, post-treatment and 8 weeks post-intervention. The primary outcome measure is comfortable gait speed as measured by the five-metre walk test [54]. Following stroke, gait speed is frequently reduced compared to age matched normals [5,55,56]. Gait speed has been shown to be reliable (r = 0.94)[57], responsive to change (SRM = 1.22; effect size = 0.83) [54], and significantly related to independent community ambulation [11].

Secondary outcome assessment will include measures of maximal gait speed, walking endurance (Six Minute Walk Test), dynamic balance (Functional Balance Test) [58], balance and walking related self-efficacy (Activities- specific Balance Confidence Scale) [59], walking function (modified Functional Ambulation Categories) [60], walking participation (5-day daily step activity - StepWatch 3 step activity monitor) [61,62], community reintegration (Life Space Questionnaire) [63,64], health related quality of life (Stroke Impact Scale 3.0) [65], goal attainment (Patient Specific Functional Scale) [66] and mean number of trainers per training session.

In addition, the baseline assessment will include the collection of demographic information, assessment of cognitive function (Mini Mental Status Exam) [36], presence of depression (Geriatric Depression Scale -15) [67], and the Chedoke-McMaster Stroke Assessment Leg and Foot stages of motor recovery [68]. At follow-up, information will be collected regarding participation in physiotherapy and any change in health status. This information will be used to describe the groups and interpret the results of the interventions.

Training and assessor therapists will record any of the following adverse events that occur during or between sessions: 1) falls (unintentionally landing on the ground), 2) any injury during session, 3) myocardial infarction (confirmed by physician and/or health records), 4) new stroke or transient ischemic attack (confirmed by physician and/or health records), 5) hospitalization for any cause, 6) death of any cause.

Physiotherapists trained to perform the standardized outcome measures will measure outcomes. Assessors will be blinded to the participant's intervention assignment and study hypotheses, limiting the potential for expectation bias. Participants will be instructed not to reveal their group assignment to the assessor.

Outcome assessment domains, tools and timing are summarized in Table 2.

**Table 2:** Outcome domains, measures and timing of assessments

*ICF*	*Domain*	*Instrument*	*Screening/Baseline*	*Post -Intervention*	*Follow-up*
Personal and Environmental Factors	Stroke details	Interview, health record review	X		
	Comorbidities		X		
	Living situation		X		
	Gait aid		X	X	X
	Physiotherapy		X	X	X
	Fall history		X	X	X
	Adverse events		X	X	X
Body Structures/Function	Motor recovery	Chedoke-McMaster Stroke Assessment	X		
	Cognition	Mini Mental Status Examination	X		
	Depression	Geriatric Depression Scale Short form-15	X		
Activity	Walking speed	5 metre walk test	X	X	X
	Walking endurance	Six Minute Walk Test	X	X	X
	Dynamic balance	Functional Balance Test	X	X	X
	Balance self-efficacy	Activities-specific Balance Confidence Scale	X	X	X
	Goal attainment	Patient Specific Functional Scale	X	X	X
Participation	Walking independence	Modified Functional Ambulation Categories	X	X	X
	Daily walking activity	Step Watch 3.0 step activity monitor	X	X	X
	Mobility participation	Life Space Questionnaire	X	X	X
	Health related quality of life	Stroke Impact Scale 3.0	X	X	X

ICF = International Classification of Function domains

### Sample size

Seventy participants will be recruited. The sample size has been calculated to reliably detect a 0.14 m/s between-group difference in gait speed change (assuming a standard deviation of 0.19 m/s) with 80% power at a 2-tail significance level of 0.05. Using self-selected gait speed as the primary outcome, this sample size has been estimated based on a range of change scores and standard deviation values reported in the literature. Reported differences in change scores between experimental and control interventions range from 0.9 m/s to 0.14 m/s and standard deviation in change scores range from 0.14 to 0.19 m/s [19,33,34,69,70]. Using a conservative estimate of standard deviation of change score of 0.19 and a difference between group change scores of 0.14 m/s, the minimal number of participants required for each treatment group is 29 participants. Dropout rates in previous studies have ranged from 7 to 20% [19,33]. Allowing for a 17% loss to follow up rate, the study will need to recruit approximately 35 participants into each group for a total of 70.

### Statistical Analysis

The trial results will be reported in accordance with the CONSORT criteria [http://www.consort-statement.org]. The flow of patients in the trial will be summarized using a flow-diagram. The baseline characteristics and outcomes scores of the patients will be analyzed using descriptive statistics reported by group as mean (standard deviation [SD]) or median (first quartile [Q1], third quartile [Q3]) for continuous variables depending on the distribution and count (percent) for categorical variables. Intention to treat analysis technique will be used for the primary analysis [71]. Missing data will be handled through multiple imputation technique [72]. All statistical tests will be performed using two-sided tests at the 0.05 level of significance. The Tukey's HSD method will be used to adjust the level of significance for testing for secondary outcomes. For all models, the results will be expressed as estimate of mean difference (or odds ratios for binary outcomes), standard errors, corresponding two-sided 95% confidence intervals and associated p-values. P-values will be reported to three decimal places with values less than 0.001 reported as < 0.001. Adjusted analyses will be performed using regression techniques to investigate the residual impact of key baseline characteristics on the outcomes (i.e. age, time since stroke onset, comfortable gait speed, and training site). Goodness-of-fit will be assessed by examining the residuals for model assumptions and chi-squared test of goodness-of-fit. All analyses will be performed using SPSS version 16.0 for Windows or SAS 9.2 (Cary, NC).

#### Primary Analysis

The post-intervention (T2) self-selected overground walking speed for the MLWP and BWSTT groups will be compared using analysis of covariance. The two factors will be intervention group (intervention or control) and baseline speed stratum (i.e. slow or fast).

#### Secondary Analysis

Mixed design analysis of variance will be used to compare the two groups' baseline, post-intervention and follow-up scores on all other secondary measures. The two factors will be time and group. Descriptive statistics (i.e. means, or frequencies) will be used to present data related to adverse and serious adverse events by groups. Any apparently significant differences between groups will be analysed for significance using chi square statistics. In an effort to describe the two interventions, the mean number of steps taken per session will be counted in a convenient sub-sample of participants using the step activity monitors. Independent samples t-test statistic will be used to compare the mean number of steps taken per session by the two groups during treatment sessions.

#### Sensitivity Analyses

Sensitivity analyses will be performed to assess the robustness of the results. First, there is likely to be high inter-correlations among all outcomes. We will use multivariate analysis of variance (MANOVA) approach to analyze all outcomes simultaneously. This method accounts for possible correlations among all outcomes and provides for a global assessment of differences between groups with an indication of where differences exist. Second, we will use generalized estimating equations (GEE) [73] to account for possible serial correlation of measurements within a patient overtime. Unlike ordinary linear regression, GEE allows accounting possible correlation of outcome scores for the same patient over time. We will use sensitivity analysis to explore potential clustering of measurements/outcomes from the same patient. The clustering effect, measured by intra-class correlation coefficient, will be assumed to be equal across patients. Sensitivity analysis will also include a between-group comparison of post-intervention comfortable gait speed in participants who completed at least 12 of the 15 training sessions using analysis of variance. Refer to Table 3 for a summary of the planned analyses.

**Table 3:** Summary of planned primary, secondary and sensitivity analyses

*Objective/Variable*	*Hypothesis*	*Outcome measure (type)* *[continuous (c), binary (b)]*	*Method of Analysis*
**1) Primary**			
Walking speed at post-intervention (T2)	MLWP > BWSTT	Comfortable gait speed (c)	ANCOVA
**2) Secondary (T2, T3)**			
Secondary outcomes			
a) Fast walking speed	MLWP > BWSTT	Fast Gait Speed (c)	ANCOVA
b) Walking endurance	MLWP > BWSTT	Six minute walk test(c)	ANCOVA
c) Balance and walking related self-efficacy	MLWP > BWSTT	Activities-specific Balance Confidence Scale (c)	ANCOVA
d) Dynamic balance	MLWP > BWSTT	Functional Balance Test(c)	ANCOVA
e) Mobility participation	MLWP > BWSTT	Life Space Questionnaire (c)	ANCOVA
f) Health-related quality of life	MLWP > BWSTT	Stroke Impact Scale 3.0 (c)	ANCOVA
g) Goal attainment	MLWP > BWSTT	Patient Specific Function Scale(c)	ANCOVA
h) Walking participation	MLWP < BWSTT	Mean daily step activity	ANCOVA
i) Training staff requirement	MLWP < BWSTT	Total number of trainers/number of training sessions (c)	T-test
j) Meaningful change in gait speed of ≥ 0.14 m/s	MLWP > BWSTT	Comfortable gait speed change score T2-T1 ≥ 0.14 m/s)(b)	Chi-square test
Adverse events (count)			
a) Falls during session		Therapist report (b)	Chi-square test
b) Injury during session		Therapist report (b)	Chi-square test
c) Falls between session		Patient report (b)	Chi-square test
d) Myocardial Infarction		Patient report/health record (b)	Chi-square test
e) New stroke		Patient report/health record (b)	Chi-square test
f) Hospitalization		Patient report/health record (b)	Chi-square test
g) Death (all causes)		Health record/Physician (b)	Chi-square test
**3) Sensitivity Analysis**			
a) All outcomes analysed simultaneously to account for correlation among them		Primary and secondary outcomes	MANOVA
b) Serial correlation of all outcomes at baseline, T2, T3		Primary and secondary outcomes	GEE
c) Completers (≥ 12 sessions)	MLWP > BWSTT	Comfortable Gait speed	ANCOVA

IMPORTANT REMARKS:

The GEE^2 ^is a technique that allows to specify the correlation structure between patients within a hospital and this approach produces unbiased estimates under the assumption that missing observations will be missing at random. An amended approach of weighted GEE will be employed if missingness is found not to be at random^3^.

In all analyses results will be expressed as coefficient, standard errors, corresponding 95% and associated p-values. Goodness-of-fit will be assessed by examining the residuals for model assumptions and chi-squared test of goodness-of-fit

Tukey's HSD method will be used to adjust the overall level of significance for multiple secondary outcomes

^1^Perera S, Mody SH, Woodman RC, Studenski SA. **Meaningful change and responsiveness in common physical performance measures**. *Journal of American Geriatrics Society *2006. **54**: 743-749.

^2^Hardin JW. *Generalized Estimating Equations*. New York: Chapman and Hall/CRC, 2001

^3 ^Diggle PJ, Liang K-Y, Zeger S. *Analysis of Longitudinal Data*. Oxford: Oxford Science Publications, 1994.

## Discussion

To date, a number of controlled trials have tested the effectiveness of intensive, task-related walking training interventions against a non-walking focused control treatment.

A head-to-head comparison of two different active walking focused interventions will help answer the question whether it matters *how *individuals practice walking after stroke. As with most rehabilitation interventions, task-related walking training can be complex and multifaceted. A sound theory base can help focus an intervention on the proposed, relevant active ingredients [74]. In our study, the experimental and comparison intervention were designed based on two different theoretic frameworks. While both interventions emphasize walking practice, their respective theory bases dictate what type of walking is practiced, the practice environment, tolerance for error and variability during practice, the role of the therapist, and the role of the participant during practice. As a result, this study provides a direct comparison of the effectiveness of two quite different task-related walking training protocols with different resource requirements. The results of this study takes an important step toward informing clinicians, patients, caregivers and administrators of the essential components of an optimally effective task-related walking training intervention following stroke.

## Competing interests

The authors declare that they have no competing interests.

## Authors' contributions

VGD conceived of the study, acted as the principal investigator and drafted the manuscript. LT provided statistical expertise in clinical trial design. All authors contributed to the study design, are grant holders, contributed to the editing of the manuscript, and read and approved the final manuscript.

## Pre-publication history

The pre-publication history for this paper can be accessed here:

http://www.biomedcentral.com/1471-2377/11/129/prepub

## References

[B1] World Health Organization The Global Burden of Disease: World Health Organization 2008 http://www.who.int/healthinfo/global_burden_disease/GBD_report_2004update_full.pdf

[B2] Jorgensen H Nakayama H Raaschou H Olsen T Recovery of walking function in stroke patients: The Copenhagen Stroke Study Archives of Physical Medicine and Rehabilitation 1995 76 27 32 10.1016/S0003-9993(95)80038-7 7811170

[B3] Pound P Gompertz P Ebrahim S A patient-centred study of the consequences of stroke Clinical Rehabilitation 1998 12 4 338 47 10.1191/026921598677661555 9744669

[B4] Moreland J DePaul V DeHueck A Pagliuso S Yip D Pollock B Wilkins S Needs assessment of individuals with stroke after discharge from hospital stratified by acute Functional Independence Measure score Disability and Rehabilitation 2009 31 26 2185 95 10.3109/09638280902951846 19903128

[B5] Olney S Richards C Hemiparetic gait following stroke. Part I: Characteristics Gait and Posture 1996 4 136 48 10.1016/0966-6362(96)01063-6

[B6] Mayo NE Wood-Dauphinee S Ahmed S Gordon C Higgins J McEwen S Salbach N Disablement following stroke Disability and Rehabilitation 1999 21 5/6 258 68 10381238 10.1080/096382899297684

[B7] Botner E Miller W Eng J Measurement properties of the Activities-specific Balance Confidence Scale among individuals with stroke Disability and Rehabilitation 2005 27 4 156 63 10.1080/09638280400008982 15824045

[B8] Salbach N Mayo N Robichaud-Ekstrand S Hanley J Richards C Wood-Dauphinee S Balance self-efficacy and its relevance to physical function and perceived health status after stroke Archives of Physical Medicine and Rehabilitation 2006 87 364 70 10.1016/j.apmr.2005.11.017 16500170

[B9] Mayo NE Wood-Dauphinee S Cote R Carlton J Durcan L Activity, Participation and Quality of Life 6 months poststroke Archives of Physical Medicine and Rehabilitation 2002 83 1035 42 10.1053/apmr.2002.33984 12161823

[B10] Lord S McPherson K McNaughton H Rochester R Weatherall M Community ambulation after stroke: how important and obtainable is it and what measures appear predictive? Archives of Physical Medicine and Rehabilitation 2004 85 234 9 10.1016/j.apmr.2003.05.002 14966707

[B11] van de Port I Kwakkel G Lindeman E Community ambulation in patients with chronic stroke: How is it related to gait speed? Journal of Rehabilitation Medicine 2008 40 23 7 10.2340/16501977-0114 18176733

[B12] Bohannon R Williams Andrew A Smith M Rehabilitation goals in patients with hemiplegia International Journal of Rehabilitation Research 1988 11 181 3 10.1097/00004356-198806000-00012

[B13] Bogey R Hornby T Gait training strategies utilized in poststroke rehabilitation: Are we really making a difference? Topics in Stroke Rehabilitation 2007 14 6 1 8 10.1310/tsr1406-1 18171655

[B14] Lindsay P Bayley M Hellings C Hill M Woodbury E Phillips S Canadian Best Practice Recommendations for Stroke Care Canadian Medical Association Journal 2008 179 12 Supplement E1 E93 10.1503/cmaj.081536 PMC2585121 19047599

[B15] Foley N Teasell R Bhogal S Mobility and the lower extremity. Evidence-based review of stroke rehabilitation http://www.ebrsr.com/reviews_details.php?Mobility-and-the-Lower-Extremity-15

[B16] Wevers L van de Port I Vermue M Mead G Kwakkel G Effects of Task-Oriented Circuit Class Training on Walking Competency After Stroke: A Systematic Review Stroke 2009 40 2450 9 10.1161/STROKEAHA.108.541946 19461035

[B17] Moore J Roth E Killian C Hornby T Locomotor training improves daily stepping activity and gait efficiency in individuals poststroke who have reached a "plateau" in recovery Stroke 2010 41 129 35 10.1161/STROKEAHA.109.563247 19910547

[B18] Miller EW Quinn ME Seddon PG Body weight support treadmill and overground ambulation training for two patients with chronic disability secondary to stroke Physical Therapy 2002 82 53 61 11784278 10.1093/ptj/82.1.53

[B19] Salbach N Mayo NE Wood-Dauphinee S Hanley J Richards C Cote R A task-orientated intervention enhances walking distance and speed in the first year post stroke: a randomized controlled trial Clinical Rehabilitation 2004 18 509 19 10.1191/0269215504cr763oa 15293485

[B20] French B Thomas LH Leathley MJ Sutton CJ McAdam J Forster A Langhorne P Price CIM Walker A Watkins CL Repetitive task training for improving functional ability after stroke Cochrane Database of Systematic Reviews 2007 4 10.1002/14651858.CD006073.pub2 17943883

[B21] Smith LN James R Barber M Ramsay S Gillespie D Chung C Rehabilitation of patients with stroke: summary of SIGN guidance British Medical Journal 2010 340 1356 8 10.1136/bmj.c2845 20551122

[B22] Hesse S Werner C von Frankenberg S Bardeleben A Treadmill training with partial body weight support after stroke Physical Medicine and Rehabilitation Clinics of North America 2003 14 S111 S123 10.1016/S1047-9651(02)00061-X 12625641

[B23] Carr JH Shepherd RB A motor relearning programme for stroke 1982 1 Oxford: William Heinemann Medical Books

[B24] Dean C Richards C Malouin F Task-related circuit training improves performance of locomotor tasks in chronic stroke: A randomized controlled pilot trial Archives of Physical Medicine and Rehabilitation 2000 81 409 17 10.1053/mr.2000.3839 10768528

[B25] Mudge S Barber PA Stott S Circuit-based rehabilitation improves gait endurance but not usual walking activity in chronic stroke: A randomized controlled trial Archives of Physical Medicine and Rehabilitation 2009 90 1989 96 10.1016/j.apmr.2009.07.015 19969159

[B26] Dietz V Body weight supported gait training: From laboratory to clinical setting Brain Research Bulletin 2009 78 I VI 10.1016/S0361-9230(08)00410-3 19070780

[B27] Dietz V Spinal cord pattern generators for locomotion Clinical Neurophysiology 2003 114 1379 89 10.1016/S1388-2457(03)00120-2 12888019

[B28] Behrman A Bowden M Nair P Neural Plasticity after spinal cord injury and training: An emerging paradigm shift in rehabilitation and walking recovery Physical Therapy 2006 86 10 1406 25 10.2522/ptj.20050212 17012645

[B29] Yen C-L Wang R-Y Liao K-K Huang C-C Yang Y-R Gait training-induced change in corticomotor excitability in patients with chronic stroke Neurorehabilitation and Neural Repair 2008 22 22 30 17507641 10.1177/1545968307301875

[B30] Forrester L Wheaton L Luft A Exercise-mediated locomotor recovery and lower-limb neuroplasticity after stroke Journal of Rehabilitation Research and Development 2008 45 2 205 20 10.1682/JRRD.2007.02.0034 18566939

[B31] Harvey R Improving poststroke recovery: Neurorecovery and task-oriented training Current Treatment Options in Cardiovascular Medicine 2009 11 251 9 10.1007/s11936-009-0026-4 19433020

[B32] Hesse S Gait training after stroke: a critical reprisal Annales de réadaptation et de médecine physique 2006 49 621 4 10.1016/j.annrmp.2006.08.002 16997413

[B33] Sullivan K Knowlton B Dobkin B Step training with body weight support: effect of treadmill speed on practice paradigms on poststroke locomotor recovery Archives of Physical Medicine and Rehabilitation 2002 83 683 91 10.1053/apmr.2002.32488 11994808

[B34] Sullivan KJ Brown DA Klassen T Mulroy S Ge T Azen SP Winstein CJ Effects of task-specific locomotor and strength training in adults who were ambulatory after stroke: results of the STEPS randomized clinical trial Physical Therapy 2007 87 1580 602 10.2522/ptj.20060310 17895349

[B35] Schmidt R Lee T Motor Control and Learning: A Behavioural Emphasis 2011 5 Champaign, IL: Human Kinetics

[B36] Crum R Anthony J Bassett S Folstein M Population-based norms for the min-mental state examination by age and educational level Journal of the American Medical Association 1993 18 2386 91 8479064

[B37] Wang D Bakhai A Wang D, Bakhai A Randomization Clinical Trials: A practical guide to design, analysis, and reporting 2006 1 London: Remedica 65 73

[B38] Meinert CL Meinert CL, Tonascia S Randomization and the mechanics of treatment masking Clinical Trial Design, Conduct and Analysis 1986 Oxford: Oxford University Press 90 112

[B39] Horak F Assumptions underlying motor control for neurologic rehabilitation Contemporary Management of Motor Control Problems: Proceedings of the II STEP Conference 1991 Fredericksberg: Foundation for Physical Therapy 11 28

[B40] Shumway-Cook A Patla A Stewart A Ferrucci L Ciol M Guralnik J Environmental demands associated with community mobility in older adults with and without mobility disabilities Physical Therapy 2002 82 670 81 12088464

[B41] Sacco K Cauda F D'agata F Mate D Duca S Geminiani G Reorganization and enhanced functional connectivity of motor areas in repetitive ankle movements after training in locomotor attention Brain Research 2009 1297 124 34 19703428 10.1016/j.brainres.2009.08.049

[B42] Kleim JA Jones TA Principles of experience dependent neural plasticity: Implications for rehabilitation after brain damage Journal of Speech, Language, and Hearing Research 2008 51 S225 S239 10.1044/1092-4388(2008/018) 18230848

[B43] Kwakkel G Kollen BJ Wagenaar RC Long term effects of intensity of upper and lower limb training after stroke: a randomised trial Journal of Neurology, Neurosurgery and Psychiatry 2002 72 473 9 10.1136/jnnp.72.4.473 PMC1737834 11909906

[B44] Lee T Swinnen S Serrien D Cognitive Effort and Motor Learning Quest 1994 46 328 44

[B45] Sherwood DE Lee TD Schema theory: critical review and implications for the role of cognition in a new theory of motor learning Research Quarterly for Exercise & Sport 2003 74 4 376 82 14768838 10.1080/02701367.2003.10609107

[B46] Carey JR Bhatt E Nagpal A Neuroplasticity promoted by task complexity Exercise and Sports Science Reviews 2005 33 1 24 31 15640717

[B47] Robinnett C Vondran M Functional ambulation velocity and distance requirements in rural and urban communities: a clinical report Physical Therapy 1988 68 1371 1373 10.1093/ptj/68.9.1371 3420171

[B48] Carr J Shepherd R Movement Science: Foundation for Physical Therapy in Rehabilitation 2000 2 Gaithersburg, Maryland: Aspen Publishers, Inc.

[B49] Van Vliet P Wulf G Extrinsic feedback for motor learning after stroke: What is the evidence? Disability and Rehabilitation 2006 28 13-14 831 840 10.1080/09638280500534937 16777770

[B50] Duncan PW Sullivan KJ Behrman AL Azen SP Wu SS Nadeau SE Dobkin BH Rose DK Tilson JK Protocol for the Locomotor Experience Applied Post-stroke(LEAPS) trial: a randomized controlled trial BMC Neurology 2007 7 39 10.1186/1471-2377-7-39 17996052 PMC2222229

[B51] Duysens J Van de Crommert H Neural control of locomotion; Part 1: The central pattern generator from cats to humans Gait and Posture 1998 7 131 41 10.1016/S0966-6362(97)00042-8 10200383

[B52] Pohl M Mehrholz J Ritshel C Ruckriem S Speed-dependent treadmill training in ambulatory hemiparetic stroke patients: a randomized controlled trial Stroke 2002 33 553 558 10.1161/hs0202.102365 11823669

[B53] Behrman A Lawless-Dixon A Davis S Locomotor training progression and outcomes after incomplete spinal cord injury Physical Therapy 2005 85 1356 71 16305274

[B54] Salbach N Mayo NE Higgins J Ahmed S Finch L Richards C Responsiveness and predictability of gait speed and other disability measures in acute stroke Archives of Physical Medicine and Rehabilitation 2001 82 1204 12 10.1053/apmr.2001.24907 11552192

[B55] Wade D Wood V Heller A Maggs J Langton A Hewer R Walking after stroke. Measurement and recovery over the first 3 months Scandanavian Journal of Rehabilitation Medicine 1987 19 25 30 3576138

[B56] Richards C Malouin F Dean C Gait in Stroke: Assessment and Rehabilitation Clinics in Geriatric Medicine 1999 15 4 833 55 10499938

[B57] Flansbjer U-B Holmback A Downham D Patten C Lexell J Reliability of gait performance tests in men and women with hemiparesis after stroke Journal of Rehabilitation Medicine 2005 37 75 82 10.1080/16501970410017215 15788341

[B58] Richardson J DePaul V Moreland J Cripps D Evaluation of the Functional Balance Test Pinnacles 1999 Winter 11 13

[B59] Powell L Myers A The Activities-specific Balance Confidence (ABC) Scale Journal of Gerontology: Medical Sciences 1995 50A 1 M28 M34 10.1093/gerona/50A.1.M28 7814786

[B60] Perry J Garrett M Gronley J Mulroy S Classification of walking handicap in the stroke population Stroke 1995 26 6 982 9 10.1161/01.STR.26.6.982 7762050

[B61] Coleman KL Smith DS Boone DA Joseph AW del Aquila MA Step activity monitor. Long term continuous recording of ambulatory function Journal of Rehabilitation Research and Development 1999 36 1 8 18 10659890

[B62] Macko RF Haueber E Shaughnessy M Coleman KL Boone DA Smith G Silver KH Microprocessor based ambulatory activity monitoring in stroke patients Medicine and Science in Sports and Exercise 2002 34 3 394 9 10.1097/00005768-200203000-00002 11880800

[B63] Baker PS Bodner EV Allman RM Measuring Life-Space mobility in community dwelling older adults Journal of the American Geriatric Society 2003 51 1610 4 10.1046/j.1532-5415.2003.51512.x 14687391

[B64] Peel C Baker PS Roth D Brown C Bodner EV Allman RM Assessing mobility in older adults: The UAB study of aging life-space assessment Physical Therapy 2005 85 10 1008 19 16180950

[B65] Duncan P Bode R Lai S Perera S Rasch Analysis of a new stroke-specific outcome scale: The Stroke Impact Scale Archives of Physical Medicine and Rehabilitation 2003 84 950 63 10.1016/S0003-9993(03)00035-2 12881816

[B66] Stratford P Gill C Westaway M Binkley J Assessing disability and change in individual patients: a report fo a patient specific measure Physiotherapy Canada 1995 47 258 63 10.3138/ptc.47.4.258

[B67] Sheikh RL Yesavage JA Geriatric Depression Scale(GDS). Recent evidence and development of a shorter version Clinical Gerontologist 1986 5 165 73 10.1300/J018v05n01_09

[B68] Gowland C Stratford P Ward M Moreland J Torresin W VanHullenaar S Measuring physical impairment and disability with the Chedoke-McMaster Stroke Assessment Stroke 1993 24 58 63 10.1161/01.STR.24.1.58 8418551

[B69] Ada L Dean C Hall J Brampton J Crompton S A treadmill and overground walking program improves walking in persons residing in community after stroke: a placebo-controlled, randomized trial Archives of Physical Medicine and Rehabilitation 2003 84 1486 91 10.1016/S0003-9993(03)00349-6 14586916

[B70] Perera S Mody SH Woodman RC Studenski SA Meaningful change and responsiveness in common physical performance measures in older adults Journal of the American Geriatric Society 2006 54 743 9 10.1111/j.1532-5415.2006.00701.x 16696738

[B71] Moher D Hopewell S Schulze KF Montorid V Gotzschee PC Devereaux PJ Elbourne D Egger M Altman DG CONSORT 2010 Explanation and Elaboration: updated guidelines for reporting parallel group randomised trials Journal of Clinical Epidemiology 2010 63 e1 e37 10.1016/j.jclinepi.2010.03.004 20346624

[B72] Sterne JAC White IR Carlin JB Spratt M Royston P Kenward MG Wood AM Carpenter JR Multiple imputation for missing data in epidemiological and clinical research: Potential and pitfalls British Medical Journal 2009 339 157 160 10.1136/bmj.b2393 PMC2714692 19564179

[B73] Hardin JW Generalized estimating equations 2001 New York: Chapman and Hall/CRC

[B74] Whyte J Using treatment theories to refine the designs of brain injury rehabilitation treatment effectiveness studies Journal of Head Trauma Rehabilitation 2006 21 2 99 106 10.1097/00001199-200603000-00003 16569984

